# Supplementary material for: Contrast-enhanced Micro-CT imaging of a foetal female pelvic floor reveals anatomical details
Source: PLoS One. 2025 Jun 13;20(6):e0314261. doi: 10.1371/journal.pone.0314261 (PMC12165418; doi:10.1371/journal.pone.0314261)
Supplement: S1 File — Due to the file-size, the acquired dataset at the last staining timepoint is only available online for viewing at https://webknossos.org/datasets/Institute_of_Anatomy/Foetus02_Lugol_15pct_152d_rec/view This is the dataset from which we extracted the images shown in Figs 1D, 1G and 2–5. Direct links to the regions shown in the panels are given in the figure captions. (DOCX) [file pone.0314261.s001.docx]

**S1 File**. **Full high-resolution tomographic dataset of the foetal sample.** Access via: <https://webknossos.org/datasets/Institute_of_Anatomy/Foetus02_Lugol_15pct_152d_rec/view>
